# Microarray analysis of genes associated with cell surface NIS protein levels in breast cancer

**DOI:** 10.1186/1756-0500-4-397

**Published:** 2011-10-11

**Authors:** Sasha J Beyer, Xiaoli Zhang, Rafael E Jimenez, Mei-Ling T Lee, Andrea L Richardson, Kun Huang, Sissy M Jhiang

**Affiliations:** 1 Integrated Biomedical Sciences Graduate Program, The Ohio State University, Columbus, Ohio 43210, USA; 2 Department of Physiology and Cell Biology, The Ohio State University, Columbus, Ohio 43210, USA; 3 Center for Biostatistics, The Ohio State University, Columbus, Ohio 43210, USA; 4 Department of Anatomic Pathology, Mayo Clinic, Rochester, Minnesota 55901, USA; 5 Department of Epidemiology and Biostatistics, University of Maryland, College Park, Maryland 20742, USA; 6 Department of Pathology, Brigham and Women's Hospital, Harvard Medical School Boston, Massachusetts 02115, USA; 7 Department of Biomedical Informatics, The Ohio State University, Columbus, Ohio 43210, USA

## Abstract

**Background:**

Na^+^/I^- ^symporter (NIS)-mediated iodide uptake allows radioiodine therapy for thyroid cancer. NIS is also expressed in breast tumors, raising potential for radionuclide therapy of breast cancer. However, NIS expression in most breast cancers is low and may not be sufficient for radionuclide therapy. We aimed to identify biomarkers associated with NIS expression such that mechanisms underlying NIS modulation in human breast tumors may be elucidated.

**Methods:**

Published oligonucleotide microarray data within the National Center for Biotechnology Information Gene Expression Omnibus database were analyzed to identify gene expression tightly correlated with NIS mRNA level among human breast tumors. NIS immunostaining was performed in a tissue microarray composed of 28 human breast tumors which had corresponding oligonucleotide microarray data available for each tumor such that gene expression associated *w*ith cell surface NIS protein level could be identified.

**Results and Discussion:**

NIS mRNA levels do not vary among breast tumors or when compared to normal breast tissues when detected by Affymetrix oligonucleotide microarray platforms. Cell surface NIS protein levels are much more variable than their corresponding NIS mRNA levels. Despite a limited number of breast tumors examined, our analysis identified cysteinyl-tRNA synthetase as a biomarker that is highly associated with cell surface NIS protein levels in the ER-positive breast cancer subtype.

**Conclusions:**

Further investigation on genes associated with cell surface NIS protein levels within each breast cancer molecular subtype may lead to novel targets for selectively increasing NIS expression/function in a subset of breast cancers patients.

## Background

The Na^+^/I^- ^symporter (NIS) (also known as SLC5A5, solute carrier family 5 member 5) is a transmembrane glycoprotein that uptakes iodide into the thyroid follicular cells for the biosynthesis of thyroid hormones. Accordingly, radioiodine has been used to ablate thyroid tumors and metastases. NIS is induced in the breast during lactation to accumulate iodide for the nursing infant to synthesize its own thyroid hormones [1,2]. NIS has also been detected in the majority of breast tumors, raising promise for radionuclide therapy of breast cancer [3-6]. However, only a minority of NIS-positive breast tumors had detectable radionuclide accumulation [4-6], indicating that strategies for selectively increasing cell surface NIS expression are critical for realizing radionuclide therapy of breast cancer patients.

Mechanisms underlying NIS modulation in human breast cancer are poorly understood. NIS expression is increased in breast tumors [3], suggesting that NIS expression is correlated with malignant transformation. However no biomarkers of breast cancer progression such as breast tumor subtype, hormone receptor status, or tumor grade [3,7-9] have been reported to correlate with NIS protein levels among tumors. The MCF-7 cell line is the only human breast cancer cell line with inducible endogenous NIS expression. Kogai *et al. *[10] first reported that trans-retinoic acid (tRA) induces NIS mRNA in MCF-7 cells at the transcriptional level. Moreover, a combination of tRA and hydrocortisone (tRA/H), further increases tRA-induced NIS expression/function in MCF-7 cells [11-15], most likely by increasing NIS mRNA stability [11]. While NIS induction by tRA has been observed in MCF-7 cell xenografts *in vivo *[12], normal mammary glands of mice [13], and the PyVT transgenic mouse model [12], Kogai et *al. *[11,12] stated that the dose of tRA required for maximum NIS induction in MCF-7 cell xenografts *in vivo *was ten-fold greater than the maximum tolerable tRA dose in humans. In this study, we aimed to identify biomarkers that correlate with NIS expression in order to elucidate mechanisms of NIS regulation in human breast tumors such that novel strategies for selectively increasing NIS expression/function in breast cancers patients can be developed.

## Methods

### Publicly Available Oligonucleotide Microarray Datasets

Published microarray datasets from NCBI GEO database [16] (http://www.ncbi.nlm.nih.gov/geo/) that detected genome wide expression in breast tumors were examined in our analysis: GSE3744 [17] (Affymetrix HG U133 Plus 2.0 Array); GSE10797 (unpublished, HG U133 Plus 2.0); GSE1561 [18] (Affymetrix HG U133A Array); GSE6367 [19] (Affymetrix HG U95Av.2); GSE6434 (unpublished data, Affymetrix HG U95A); GSE3155 [20] (Agilent-012391 Whole Human Genome Oligo Microarray and Applied Biosystems Human Genome Survey Microarray Version 1); GSE6861 [21] (Affymetrix Human X3P Array); GSE14548 [22](Affymetrix Human X3P Array).

### Cell Culture and RNA Extraction

MCF-7 human breast cancer cells were maintained in a 1:1 ratio of DMEM and Ham's F-12 media (Gibco), 10% FBS (Invitrogen) and 1% penicillin/streptomycin. MCF-7 cells were treated with DMSO vehicle, tRA, or tRA/H for 12 hours, and total RNA was extracted with Trizol reagent (Invitrogen) and chloroform (Sigma).

### Generation of microarray data and real-time qRT-PCR

RNA integrity and quantity were determined by the Agilent Bioanalyzer 2100 by the MicroArray Shared Resource (MASR) for the OSU Comprehensive Cancer Center (OSUCCC). The MASR performed sample preparation and labeling, chip hybridization and staining, chip scanning and initial image analysis according to Affymetrix instructions. Briefly, cRNA was generated using GeneChip T7-Oligo(dT) Promoter Primers kit and the raw data was generated with Affymetrix GeneChip Operating Software on a Human Genome U133 plus 2.0 Affymetrix platform. Raw microarray data from MCF-7 breast cancer cells treated with DMSO, tRA and tRA/H are included on the GEO database (GSE32161).

Quantitative RT-PCR was performed using Power SYBR Green PCR Master Mix (Applied Biosystems). NIS was amplified with primers: 5'-CCGGATCAACCTCATGGACT-3' and 5'-CTGAGGGTGCCACTGTAAG-3'. Human GAPDH was amplified with primers: 5'-CATCATCTCTGCCCCCTCTGCTG-3' and 5'-GCAATGCCAGCCCCAGCGTCAAAGG-3'. PCR using the ABI 7900HT instrument (Applied Biosystems) was performed by OSUCCC Nucleic Acids Shared Resource.

### Breast tumor tissue microarray with corresponding, pre-existing microarray data

A breast tumor tissue microarray composed of 28 tumors of various histological and molecular subtypes was previously described by Lu *et al. *[23] (GEO database, GSE5460). Briefly, all samples were cases of primary tumors from untreated breast cancers with known axillary lymph node status. Information on invasive tumor subtype, molecular subtype, estrogen receptor (ER) status, progesterone receptor (PR) status, Her-2/neu status, p53 status, Bloom-Richardson grade and the presence of lymph node metastases were also obtained for each tumor, as described by Lu *et al. *[23]. Each tumor had available corresponding gene expression data from the HG-U133 Plus 2.0 microarray platform.

### Immunohistochemistry

Immunohistochemistry was performed as described by our previous study [24]. Tissues were incubated with either p442 anti-hNIS (1:25) or anti-CARS (Sigma, 1:500) primary antibodies for one hour. The level of cell surface NIS protein in each case was scored on a scale of 0, 1+, 2+ and 3+, using criteria analogous to the evaluation of Her-2/neu staining. For CARS protein, tumors scored as 0 or 1+ were considered negative and tumors scored as 2 or 3+ were considered positive.

#### Statistical Analyses

##### Oligonucleotide microarray analysis

For all the microarray studies, background correction and normalization was performed and gene expression level was summarized over probes using the RMA method [25]. Normalized data sets are included on the GEO database (GSE32161). A filtering method based on the percentage of samples with expression values above noise level was applied to filter out probe-sets with little or no expression. Generalized linear models based on Limma [26] were used to detect differentially expressed genes between NIS "0" and NIS "2+ and 3+" breast tumor groups, as well as between DMSO-, tRA- or tRA/H-treated MCF-7 cells. In order to improve the estimates of variability and statistical tests for differential expression, a variance shrinkage method was employed [27]. The differentially expressed genes were claimed based on the p-values by controlling the average number of false positives at 1 [28] over all the tested probe sets. Fold changes of at least 1.5 were used to further reduce the list of significant probe sets after controlling the number of false positives. In order to detect NIS-associated genes, Spearman rank correlation and/or Pearson correlation method was applied using the normalized and filtered microarray data. Genes with absolute correlation coefficients greater than 0.6 and p-values above the significance level (controlling 1 false positive over all the tested probe-sets) were selected. Differentially expressed genes or NIS correlated genes were subjected to hierarchical clustering using Euclidean distance based on their relative expressions and average linkage clustering method using software MEV [29].

##### Bioinformatics Tools

After NIS-associated genes were identified by Linear Models, Pearson correlation and Spearman correlation analyses, bioinformatics tools were used for data analysis. Ingenuity Pathway Analysis (Ingenuity Systems) allowed us to identify cell signaling pathways or functional categories that were over-represented among the NIS-associated genes. Ingenuity Pathway Analysis also compared and contrasted gene lists identified by each statistical method, as indicated by Venn diagrams. ToppFun was also used to determine whether the identified NIS-associated genes were regulated by a common transcription factor [30].

##### Correlation studies

Fisher's exact test was used to test the association between cell surface NIS protein levels and HER-2 status, tumor type, ER status, PR status, p53 mutation status, and CARS protein.

## Results

### NIS mRNA levels detected by HG U133A oligonucleotide microarray (NIS probe set ID 211123_at) do not vary among breast tumors or when compared to normal breast tissue

**Figure 1:**
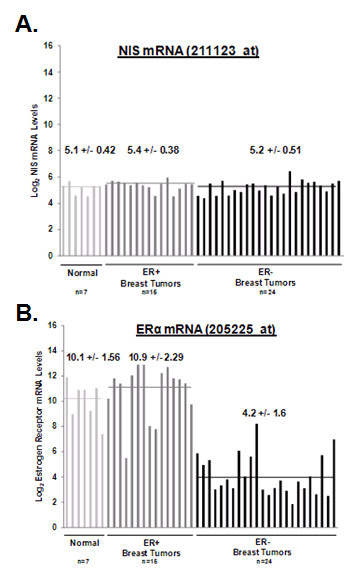
**NIS mRNA levels detected by HG U133A oligonucleotide microarray (NIS probe set ID 211123_at) do not vary among breast tumors or normal breast tissue**. The Gene Expression Omnibus dataset GSE3744 consists of normalized log_2_-transformed genome-wide expression of 7 normal breast tissues, 15 normal ER+ breast tumors and 24 ER- breast tumors. NIS and ER mRNA expression are plotted for each individual breast tissue sample and horizontal lines represent mean expression values among normal breast tissues (*light gray line*), ER+ breast tumors (*dark gray line*), and ER- breast tumors (*black line*). Mean ± standard deviation are also indicated. (A) NIS mRNA levels of breast tissues in the GSE3744 Gene Expression Omnibus dataset did not significantly vary among normal breast tissues (mean 5.1 ± 0.42, range 4.5-5.7), ER+ breast tumors (mean 5.4 ± 0.39, range 4.6-6.0) or ER- breast tumors (mean 5.2 ± 0.51, range 4.4-6.4). (B) In contrast, ER mRNA levels for normal breast tissues (mean 10.1 ± 1.56, *light gray line*; range 7.4-11.9) and ER+ breast tumors (mean 10.9 ± 2.29, *dark gray line*; range 5.5-13.9) were significantly greater than ER- tumors (mean 4.2 ± 1.6, *black line*; range 1.9-8.2).

NIS expression among breast tumors within the GSE3744 dataset [17] was detected by the 211123_at NIS probe set on the Affymetrix HG-U133 Plus 2.0. However, the 211123_at NIS probe set did not detect significant differences in NIS mRNA levels despite that not all breast tumors have been reported to express NIS mRNA [5,9,31]. As shown in Figure 1A, log_2 _NIS mRNA levels detected among normal, non-lactating breast tissues (mean 5.1 ± 0.42, *light gray line*) were comparable to breast tumor NIS mRNA levels (ER+, 5.4 ± 0.38, *dark gray line*, and ER-, 5.2 ± 0.51, *black line*). NIS mRNA levels detected by the 211123_at NIS probe set deviated by less than 10% of the mean. In comparison, as shown in Figure 1B, log_2 _ERα mRNA levels were greater in ERα-positive tissues compared to ERα-negative tumors. Overall, ER mRNA level deviated by up to 38% of the mean. Taken together, the NIS probe set may not have sufficient sensitivity to detect NIS variability among tumors and/or NIS protein levels may be primarily regulated at the post-mRNA level.

### Only modest increases in NIS mRNA expression were detected in tRA- and tRA/H-treated MCF-7 breast cancer cells by the 211123_at NIS probe set

**Figure 2:**
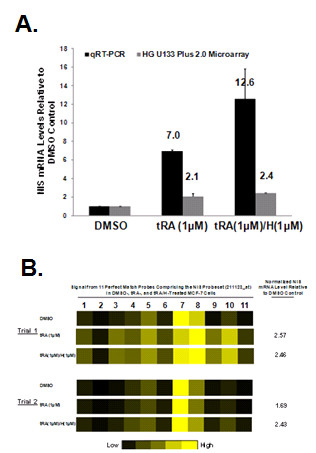
**Only modest increases in NIS mRNA expression were detected in tRA- and tRA/H-treated MCF-7 breast cancer cells by the 211123_at NIS probe set**. MCF-7 human breast cancer cells were treated with DMSO vehicle, tRA(1 μM) or tRA(1 μM)/H(1 μM) for 12 hours, total RNA was harvested, and NIS mRNA was detected by parallel qRT-PCR and microarray (HG U133 Plus 2.0) experiments. The quantification of hNIS by qRT-PCR was normalized according to the level of GAPDH and the data are presented as a fold change in NIS mRNA over GAPDH control. (A) While qRT-PCR detected 7.0- and 12.6-fold increases in NIS mRNA with tRA and tRA/H treatments, respectively, only 2.1- and 2.4-fold increases were detected by microarray. The mean ± standard deviations of two independent trials are plotted. (B) Signal intensities detected by the 11 individual perfect match probes within the NIS probe set are depicted for two independent trials of DMSO-, tRA- and tRA/H-treated MCF-7 cells. The figure depicts variability in the extent of change in NIS mRNA among all 11 probes with tRA and tRA/H treatment. Fold changes in normalized NIS mRNA levels are also indicated for each individual trial.

In agreement with previous reports [10,11], qRT-PCR detected 7-fold and 12.6-fold increases in NIS mRNA levels with tRA and tRA/H treatment of MCF-7 cells, respectively. Oligonucleotide microarray technology only detected 2.1- and 2.4-fold increases in tRA- and tRA/H-induced NIS mRNA levels, respectively (Figure 2A). Gene expression profiles of tRA-treated MCF-7 cells were compared to DMSO vehicle-treated MCF-7 cells (Additional File 1) with the objective of identifying genes contributing to tRA-induced NIS expression. However, NIS was not among the differentially expressed genes. It is important to note that many genes previously reported to be up-regulated by tRA treatment were identified, including cytochrome P450, G-protein coupled receptor, S100 calcium binding protein, LY6/PLAUR domain containing protein, lamins A and C, ceruloplasmin and transforming growth factor β [reviewed in [32]], thus validating our method of analysis. These results further indicate that the 211123_at NIS probe set is not sufficient to detect increased NIS mRNA level in tRA/H treated MCF-7 cells.

Since the 211123_at NIS probe set is composed of 11 perfect match probes, we next examined the extent of tRA- and tRA/H-induced NIS mRNA levels detected by each individual probe to determine whether this may contribute to the low sensitivity of the NIS probe set. The extent of change in signal was highly variable among the 11 probes. While most probes indicated minimal increases in signal with tRA and tRA/H treatment, one probe showed no increase in signal intensity with tRA- or tRA/H treatment (Figure 2B, *probe 2*) and another decreased in intensity (Figure 2B, *probe 7*). Selecting for individual probes that most accurately reflect variability in NIS mRNA expression may improve the detection of NIS mRNA by the 211123_at NIS probe set.

### Variability in NIS mRNA expression among breast tumors could not be detected by additional oligonucleotide microarray platforms

As shown in Table 1, NIS mRNA expression among breast tumors deviated from the mean by less than 12% in two additional datasets utilizing the HG U133 Plus 2.0 platform, confirming that the sensitivity of the 211123_at probe set was too low to detect variability in NIS mRNA levels among breast cancers. We analyzed four additional microarray platforms that detected genome-wide expression among breast tumors on the GEO database. Table 1 summarizes the NIS probe sets among these four additional microarray platforms, as they varied according to probe length, quantity of individual perfect match probes, and target sequence within the 3' region of NIS cDNA. NIS mRNA level deviated by less than 12% of the mean regardless of the NIS probe set/microarray platform utilized, suggesting that NIS variability among breast cancers cannot be reliably detected by several Affymetrix platforms. Moon et al. [5] previously reported that nearly all breast tumors examined (N = 24/25) had detectable NIS mRNA by RT-PCR and the extent of variation in NIS mRNA levels among tumors was about 10-fold (median 0.6 ± 0.27, range 0.10-1.27 arbitrary units).

**Table 1:** NIS mRNA levels do not vary among human breast tumors as detected by oligonucleotide microarray technology

Microarray Platform	NIS Probe Set ID	Probe Length	Target (NIS Sequence)	Standard Deviation
Affymetrix HG U133 Plus 2.0	211123_at	25-mer (n* = 11)	5'-1521-1991-3'	Avg. 0.23, Range 0.17-0.28 (N^# ^= 2)
Affymetrix HG U95A	32459_at	25-mer (n* = 16)	5'-1522-1998-3'	Avg. 0.20, Range 0.14-0.26 (N^# ^= 2)
Affymetrix Human X3P	G2887404_3p_at	25-mer (n* = 11)	5'-1693-1992-3'	Avg. 0.18, Range 0.18-0.18 (N^# ^= 2)
Applied Biosystems Human Survey Microarray V1	193100	60-mer (n* = 1)	Proprietary	0.21 (N^# ^= 1)
Agilent-012391 Human Genome	30047	60-mer (n* = 1)	5'-1707-1767-3'	0.13 (N^# ^= 1)

*, Number of perfect match oligonucleotide probes within the NIS probe set.

#, Number of datasets examined that utilized each respective microarray platform.

### Cell surface NIS protein levels are more variable than corresponding NIS mRNA levels detected by oligonucleotide microarray

To identify gene expression profiles associated with cell surface NIS protein levels, NIS-immunostaining was performed using a TMA with cDNA microarray data available for each breast tumor (GEO dataset GSE5460)[23]. Two cores extracted from different regions of each tumor were included on the TMA and independently scored by three individuals, including an experienced breast cancer pathologist (R.J.). Among the tumors on the TMA, 5 (18%) were scored 0, 6 (21%) were 0/1+, 7 (25%) were 1+, 5 (18%) were 1/2+, 3 (11%) were 2+, and 2 (7%) were assigned a score of 3+. Similar to our previous study [24], only 7% (3+) to 18% (2-3+) of breast tumors strongly expressed NIS protein and 18% (0+) of breast tumors were considered negative for NIS protein. Also consistent with previous reports [3,7-9], there was no correlation between cell surface NIS protein and breast tumor subtype (p = 0.50), hormone receptor status (ER, p = 0.17; PR, p = 0.58; Her-2/neu, p = 0.97), p53 status (p = 0.71), tumor grade (p = 0.57), or the presence of lymph node metastases (p = 0.59) (Table 2).

**Table 2:** Summary of cell surface NIS protein levels and characteristics of 28 breast tumors included on the TMA

Tumor ID	Cell surface NIS protein	Molecular Subtype	Invasive Tumor Type	Bloom-Richardson Grade	PR Status	ER Status	Her-2/neu Status	P53 Status	Lymph Node Metastases
1	0	Basal	D	III	-	-	-	-	-
2	0	Basal	D	III	-	-	-	-	-
3	0	ER+	D	II	+Low	+	-	+	+
4	0	HER2/neu	D	III	-	+	+	-	+
5*	0	HER2/neu	M	II	+	+	+	-	+
6*	0/1	HER2/neu	D	III	-	-	+	+	+
7	0/1	ER+	D	III	+	+	+	Unknown	-
8	0/1	ER+	L	I	-	+	-	-	+
9	0/1	ER+	D	III	+	+	-	+	+
10	0/1	HER2/neu	L	III	+	+	+	Unknown	+
11	0/1	ER+	L	II	+	+	-	-	+
12	1+	HER2/neu	D	III	-	-	+	+	-
13	1+	Basal	D	III	-	-	-	+	+
14	1+	HER2/neu	D	III	-	-	+	+	+
15	1+	HER2/neu	D	III	-	-	+	-	+
16	1+	ER+	L	I	+	+	-	Unknown	+
17*	1+	ER+	L	II	+Low	+	-	+Low	+
18*	1+	HER2/neu	D	III	+Low	+	-	+Low	Unknown
19	1+/2+	HER2/neu	D	III	-	-	+	-	-
20	1+/2+	Basal	D	III	-	-	-	+	-
21	1+/2+	Basal	D	III	-	-	-	+	-
22	1+/2+	Basal	M	III	-	-	+	+	+
23	1+/2+	ER+	D	III	+	+	-	Unknown	+
24	2+	ER+	D	II	-	-	-	-	-
25	2+	ER+	D	II	+	+	-	-	+
26	2+	HER/neu	L	III	-	-	+	+	+
27	3+	Basal	D	III	-	-	-	+	+
28	3+	Basal	D	III	-	-	-	-	+

*: Tumors not included in microarray analysis due to microarray data quality; +: Positive; +Low: Positive-Low; -: Negative; D: ductal carcinoma; L: Lobular carcinoma; M: Mixed ductal and lobular carcinoma; ER: estrogen receptor.

**Figure 3:**
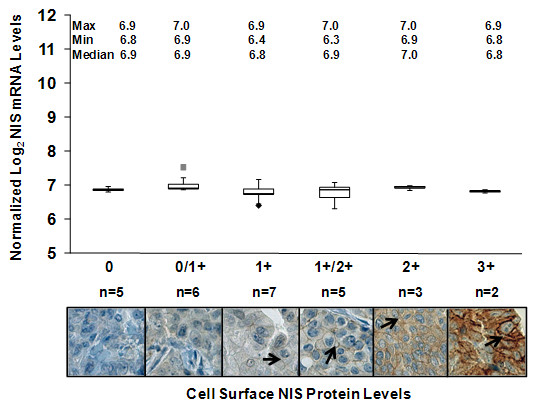
**Cell surface NIS protein levels are more variable than corresponding NIS mRNA levels detected by oligonucleotide microarray**. A human breast tumor TMA composed of 28 breast tumors with corresponding cDNA microarray data was immunostained with the p442 anti-hNIS antibody and each tumor was evaluated according to the level of cell surface NIS protein on a scale of 0, 0/1+, 1+, 1+/2+, 2+ and 3+. NIS mRNA levels detected by the 211123_at NIS probe set of the HG U133 Plus 2.0 Affymetrix microarray platform were examined and compared among breast tumors expressing each assigned level of cell surface NIS protein. Maximum, minimum and median NIS mRNA levels for each level of cell surface NIS are indicated. As shown by the box and whisker plot, there was no correlation between NIS mRNA and cell surface NIS protein among breast tumors. The length of the box represents the interquartile range (i.e., the middle 50% of the data). The median (*line through the middle of each box*), the lower quartile (*bottom line of each box*), and the upper quartile (*top line of each box*) are also specified on the plot for each level of cell surface NIS protein. The sample minimum and maximum values are represented as T-shaped lines extending from the ends of the box. Maximum outliers (*gray squares*) and minimum outliers (*black diamonds*) are also plotted. Representative images of breast tumors scored as 0 (18%, n = 5), 0/1+ (21%, n = 6), 1+ (25%, n = 7), 1+/2+ (18%, n = 5), 2+ (11%, n = 3) and 3+ (7%, n = 2) for cell surface NIS protein (*denoted by arrows*) are also shown (400×).

As expected, there was no correlation between NIS mRNA levels detected by the 211123_at NIS probe set and cell surface NIS protein levels (Figure 3). Indeed, NIS mRNA levels did not vary between cell surface NIS-negative tumors (0+) and cell surface NIS-positive tumors (3+). Thus, identifying gene expression profiles associated with cell surface NIS protein may be a more effective approach than identifying gene expression profiles associated with NIS mRNA levels.

### Linear Models Analysis identified 44 genes to be significantly up- or down-regulated in cell surface NIS-positive breast tumors compared to cell surface NIS-negative breast tumors

Considering the inherent limitations and subjectivity associated with quantifying cell surface NIS protein levels by immunohistochemical staining, we initially identified gene expression profiles associated with NIS protein by comparing tumors that were strongly positive for cell surface NIS (2+, n = 3; 3+, n = 2) and negative for cell surface NIS (0+, n = 4). Forty four genes were significantly differentially regulated according to cell surface NIS protein level by Linear Models analysis, with 42 up-regulated and 2 down-regulated in NIS-positive breast tumors compared to NIS-negative breast tumors.

Clustering analysis of the 44 identified genes appears to group tumors based on their molecular subtypes rather than distinguishing NIS-positive tumors from NIS-negative tumors (Additional File 2: Figure A). However, the distinction in gene expression profiles between NIS-positive basal tumors and NIS-negative basal tumors was readily apparent by the heat map (*breast tumor ID 1, 2 vs. 27, 28*). Indeed, scatter plots show that mRNA levels of IGFBP2 and SPIB, the two most differentially regulated genes, were significantly different between NIS-negative (0+) versus NIS-positive (3+) breast tumors (Additional File 2: Figure B). Taken together, while the power of this Linear Models analysis was limited by the small number of breast tumors examined, this analysis suggests that biomarkers associated with cell surface NIS expression may vary according to breast cancer molecular subtypes.

### Pearson and Spearman correlation analyses identified genes positively- and negatively-correlated with cell surface NIS protein levels in breast cancer

Pearson correlation analysis identified 63 genes that exhibited a linear correlation (r ≥ 0.6) with cell surface NIS levels among the 24 breast tumors. However, the cluster analysis did not appear to group breast tumors according to molecular subtype or cell surface NIS levels (Additional File 3: Figure A). Since Pearson correlation is more susceptible to outliers, Spearman rank correlation was also performed to identify gene expression that was monotonically correlated with cell surface NIS levels. Spearman rank correlation identified 64 genes, and the cluster analysis appeared to group tumors according to cell surface NIS protein level rather than molecular subtype (Additional File 3: Figure B).

NIS gene was not included among the positively correlated genes identified by either analysis, most likely due to the insensitivity of the 211123_at NIS probe set. No over-represented cell signaling pathways or functional categories of genes were identified by Ingenuity Pathway Analysis (Ingenuity Systems) and no transcription factors commonly associated with the identified genes were found by ToppFun (ToppGene Suite)[30]. There were 30 genes in common between Pearson and Spearman rank correlation analyses, suggesting that at least 47% of the 64 genes identified by Spearman correlation exhibited linear relationships with cell surface NIS levels. Scatter plots showed that both QRICH1 and ND6 mRNA levels appeared to have linear relationships with cell surface NIS protein levels, however, the extent of variation in QRICH1 mRNA level according to NIS cell surface levels is limited (Additional File 3: Figure C).

### Pearson correlation, Spearman rank correlation, and Linear Models analyses identified CARS and PYROXD1 to be highly associated with cell surface NIS protein in breast cancer

**Figure 4:**
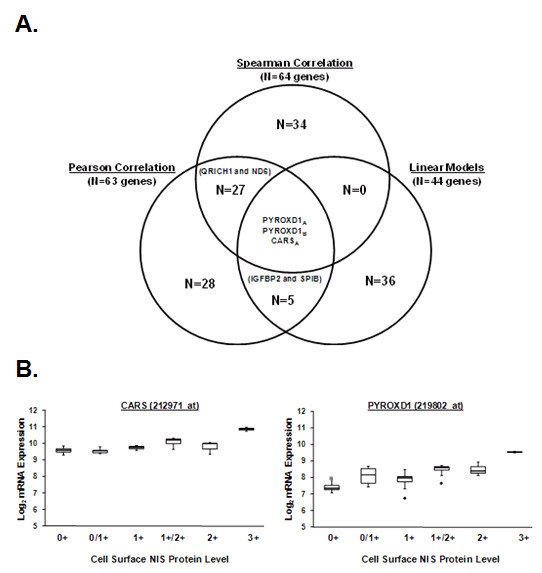
**CARS and PYROXD1 genes were identified by Pearson correlation, Spearman rank correlation, and Linear Models analyses**. (A) Overlapping genes identified by the Spearman rank correlation, Pearson correlation and Linear Models microarray analyses are indicated by the venn diagram. Two PYROXD1 probesets (*denoted as PYROXD1_A _and PYROXD1_B_*) and CARS (*probeset ID 212971_at*) were identified by all three analyses. (B) The box and whisker plot examines and compares normalized mRNA expression of CARS and PYROXD1 among breast tumors scored as 0, 0/1+, 1+, 1+/2+, 2+ or 3+ for cell surface NIS protein. As shown by the scatter plot, CARS and PYROXD1 mRNA levels correlate with cell surface NIS protein levels. The length of the box represents the interquartile range (i.e., the middle 50% of the data). The median (*line through the middle of each box*), the lower quartile (*bottom line of each box*), and the upper quartile (*top line of each box*) are also specified on the plot for each level of cell surface NIS protein. The sample minimum and maximum values are represented as T-shaped lines extending from the ends of the box. Maximum outliers (*gray squares*) and minimum outliers (*black diamonds*) are also plotted.

Genes commonly identified by Pearson correlation, Spearman rank correlation, and Linear Models analyses were further examined. As shown by the Venn diagram in Figure 4A, cysteinyl-tRNA synthetase (CARS) and two different probe sets targeting pyridine nucleotide-disulphide oxidoreductase domain (PYROXD1) were identified by each statistical analysis. Scatter plots showed that CARS and PYROXD1 were not only highly correlated with cell surface NIS protein level but also differentially regulated between NIS-positive and NIS-negative breast tumors (Figure 4B).

### CARS protein is associated with cell surface NIS protein among ER+ breast tumors

**Figure 5:**
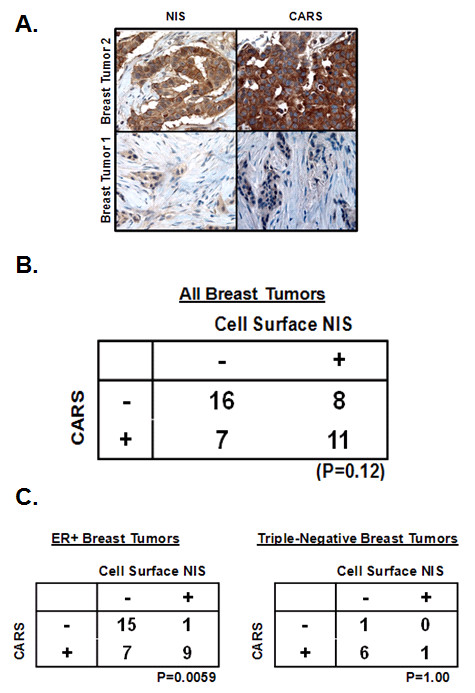
**CARS protein is associated with cell surface NIS protein among ER+ breast tumors**. Cell surface NIS and CARS protein were probed by immunohistochemistry on another breast tumor TMA composed of 42 tumors of various histological subtypes and tumors were assigned a score of 0, 1+, 2+ and 3+ for each respective protein. Scores of 0 and 1+ were considered to be negative and scores of 2+ and 3+ were considered positive. (A) Positive and negative NIS and CARS immunostaining from two representative breast tumors are shown. (B) While there appeared to be a trend of over- or under-expression of CARS in all NIS positive and negative tumors, respectively, the correlation did not achieve statistical significance by Fisher's exact test (P > 0.05). (C) Interestingly, CARS protein significantly correlated with breast tumors of ER+ histological subtype but not tumors triple negative for ER, PR and Her-2/neu.

Immunostaining was performed on a different TMA enriched for NIS-positive tumors (42 tumors, including 19 NIS-positive tumors (2+/3+) and 23 NIS-negative tumors (0+/1+) to confirm the results of our oligonucleotide microarray analysis. Unfortunately, there was no antibody available for confirmatory experiments involving PYROXD1, as this gene codes for the pyridine nucleotide-disulphide oxidoreductase domain, a domain found among proteins involved in disulphide bond formation [33]. As shown in Figures 5A and 5B, CARS showed a pattern of over- or under-expression in NIS-positive or negative tumor groups, respectively, consistent with our oligonucleotide microarray data. However, this trend did not reach statistical significance among all 42 breast tumors (p > 0.05). It was not until cell surface NIS and CARS protein levels were examined in only ER+ breast tumors (n = 32) that the association between cell surface NIS and CARS reached statistical significance (Figure 5C, p < 0.05). In contrast, triple-negative breast tumors (ER-, PR-, and Her-2/neu-, n = 8) showed no relationship between cell surface NIS protein and CARS protein (Figure 5C, p > 0.05), again indicating that biomarkers of NIS protein level may vary among different breast cancer subtypes.

## Discussion

NIS variability among breast cancers could not be reliably detected by any one of multiple Affymetrix platforms included in our study. Instead, we identify CARS as a biomarker that is correlated with cell surface NIS levels among ER+ breast cancers. Our analysis indicates that the underlying mechanism of NIS modulation is most likely to be different among different subtypes of breast cancer. Gene expression profiling for biomarkers associated with cell surface NIS protein levels among breast cancer subtypes with a larger sample number is warranted. Biomarkers identified will not only help to recognize subsets of breast cancer patients likely to benefit from NIS-targeted radionuclide therapy, but may also serve as novel targets for developing strategies to selectively increase NIS-mediated radionuclide uptake in corresponding breast cancer subtypes.

Microarray technology is known to underestimate fold changes in gene expression compared to qRT-PCR. Studies have reported significantly higher changes in mRNA levels detected by qRT-PCR compared to microarray technology [34], as microarray technology is based on hybridization rather than gene amplification. In some cases, saturation of microarray probes has been reported to contribute to underestimations in gene expression [35]. Moreover, while individual probes within probe sets of microarray platforms are designed to improve signal-to-noise ratio and minimize non-specific hybridization, the variability in signal intensity detected by these probes can introduce error [36]. Alternatively, NIS mRNA levels quantified by an qRT-PCR array may be better equipped to distinguish between NIS-positive and NIS-negative breast tumors for gene expression profiling, however, qRT-PCR arrays capable of detecting genome wide expression are not yet commercially available. Moreover, additional breast tumor mRNA is unfortunately not available for confirming our microarray and/or NIS immunohistochemical staining results by quantitative RT-PCR.

It is not known whether NIS is modulated at the post-mRNA level in breast cancer. It has been reported that 34-96% of breast tumors express NIS mRNA [5,9,30]. However, Moon et al. [5] was the only study to quantify NIS mRNA levels among breast tumors by competitive RT-PCR followed by densitometry of ethidium bromide staining. Interestingly, Moon et al. [5] reported that nearly all breast tumors had NIS mRNA levels comparable to that of peri-tumoral normal breast tissues. Moreover, the overlap in mRNA levels among breast tumors with and without detectable ^99m^Tc pertechnetate uptake [5] further suggests that NIS may be regulated at the post-mRNA level. Accordingly, a correlation analysis in which NIS mRNA levels detected by a more sensitive qRT-PCR assay compared with corresponding cell surface NIS protein levels among breast tissues should be performed to elucidate the extent of post mRNA NIS regulation. However, the variability in total NIS protein (combination of intracellular and cell surface NIS protein) among breast tumors remains debatable. Indeed, NIS cell surface trafficking impairments that result in the accumulation of intracellular NIS immunostaining has been suggested to occur in breast cancer [3,6]. However, our recent study [24] and Peyrottes *et al. *[37] indicated that intracellular anti-NIS antibody immunostaining may be non-specific. Finally, gene expression profiles associated with cell surface NIS levels may not only reflect differences in NIS expression, but also differences in NIS protein stability and NIS cell surface trafficking. Nevertheless, our breast cancer microarray analysis did not identify cell signaling factors previously reported to modulate NIS cell surface localization, such as the proto-oncogene PTTG-binding factor (PBF) [38] or phosphatidylinositol-3 kinase (PI3K) [7].

The biological relationship between CARS and NIS remains uncertain. Interestingly, while CARS is best known for its role in the ligation of cysteine to tRNA during protein translation, its expression has also been associated with breast cancer [39]. CARS is located on chromosome 11p15.5, one of the most highly imprinted chromosomal regions. Many genes on chromosome 11p15.5, including insulin growth factor II (IGF II), are susceptible to loss of imprinting (LOI), in which both paternal and maternal alleles are aberrantly expressed during breast cancer progression [40]. In fact, IGF II, has not only been reported to have increased expression in breast tumors compared to normal breast tissues, it has also been reported to transiently increase NIS expression in MCF-7 breast cancer cells [41]. Thus, the positive association between CARS mRNA and cell surface NIS protein in breast tumors could be contributed indirectly by the location of CARS on chromosome 11p15.5. It would therefore be interesting to further explore the relationship between CARS and NIS expression by determining whether siRNA-mediated loss of CARS function interferes with functional NIS expression in MCF-7 breast cancer cells.

Finally, the TMA selected to perform CARS immunostaining contained breast tumors categorized as ER+ or triple negative (ER-, PR- or Her-2/neu). Since triple-negative breast tumors are not synonymous with basal breast tumors [reviewed in [42]], it would be interesting to further examine whether the association between CARS and cell surface NIS protein level is restricted to ER+ breast tumors.

## Conclusions

NIS modulation among various breast tumors mainly occurs at the translational and/or post-translational levels. The cysteinyl-tRNA synthetase is highly associated with cell surface NIS protein levels in breast tumors of the ER-positive subtype. Further investigation on biomarkers correlated with cell surface NIS protein levels within each breast cancer molecular subtype may lead to tailored strategies enabling NIS-targeted radionuclide therapy for subsets of breast cancer patients.

## Competing interests

The authors declare that they have no competing interests.

## Authors' contributions

SB performed the MCF-7 cell microarray and RT-PCR experiments, analyzed the MCF-7 cell and breast tumor microarray data and helped to draft the manuscript. XZ and ML performed statistical analyses for the microarray experiments. RJ, a breast cancer pathologist, was involved in reading and scoring immunostained breast tumors for cell surface NIS levels. AR contributed the breast tumor microarray data and tissues. KH offered suggestions for the study design and analyzing data using bioinformatics approaches. SJ was involved in designing the study, supervising experiments and drafting the manuscript. All authors read and approved the final manuscript.

## Supplementary Material

Additional file 1 **Cluster analysis of 138 genes identified to be significantly up- or down-regulated with tRA treatment compared DMSO vehicle in MCF-7 cells, as determined by Linear Models analysis**. MCF-7 human breast cancer cells were treated with DMSO vehicle or tRA (1 μM) for 12 hours, total RNA was harvested, genome-wide expression was detected by oligonucleotide microarray (Affymetrix HG U133 Plus 2.0) and genome-wide expression was compared between treatments by Linear Model analysis. Significance was assigned to 138 genes with a false discovery rate ≤0.045% and a p-value less than 0.00045. The heat map shows expression of these 138 genes in tRA- and DMSO vehicle-treated MCF-7 cells from two independent trials. Twenty eight genes were significantly up-regulated and 110 genes were significantly down-regulated in tRA-treated MCF-7 cells compared to DMSO vehicle control, although the NIS gene was not identified among them. Genes with high expression are denoted in *red *and genes with low expression are denoted in *green*. No genes were commonly identified by both the MCF-7 cell model and breast tumor analyses. Click here for file

Additional file 2 **Cluster analysis of 44 genes identified to be significantly up- or down-regulated in cell surface NIS-positive breast tumors compared to cell surface NIS-negative breast tumors by Linear Models analysis**. The Linear Models analysis compared gene expression of 4 breast tumors considered to be negative for cell surface NIS protein (0+) to 5 tumors considered to be strongly positive for cell surface NIS protein (2+/3+). (A) Breast tumor IDs, molecular subtypes and cell surface NIS protein levels of breast tumors are indicated on the heat map. Significance was assigned to genes with a False Discovery Rate threshold of ≤0.038% and a p-value < 0.0004. The Linear Models analysis identified 42 genes to be significantly up-regulated and 2 genes to be significantly down-regulated in cell surface NIS-positive tumors compared to cell surface NIS-negative tumors. The cluster analysis shown in the heat map above appeared to cluster ER+ and HER-2/neu breast tumors according to molecular subtype and, within the ER+ molecular subtype, breast tumors appeared to group according to the level of cell surface NIS protein. In general, gene clusters capable of distinguishing between NIS-positive and NIS-negative ER+ and Her-2/neu breast tumors could not be identified. In contrast, cluster analysis distinguished between cell surface NIS-positive and cell surface NIS-negative basal breast tumors. High gene expression is denoted in *red *and low gene expression is denoted in *green *on the heat map. (B) The box and whisker plot examines and compares normalized mRNA expression of IGFBP2 and SPIB among breast tumors scored as 0, 0/1+, 1+, 1+/2+, 2+ or 3+ for cell surface NIS protein. The length of the box represents the interquartile range (i.e., the middle 50% of the data). The median (*line through the middle of each box*), the lower quartile (*bottom line of each box*), and the upper quartile (*top line of each box*) are also specified on the plot for each level of cell surface NIS protein. The sample minimum and maximum values are represented as T-shaped lines extending from the ends of the box. Maximum outliers (*gray squares*) and minimum outliers (*black diamonds*) are also plotted. Click here for file

Additional file 3 **Genes identified to be positively- or negatively-correlated with cell surface NIS protein levels in breast cancer**. The (A) Pearson and (B) Spearman rank correlation analyses compared gene expression of 24 breast tumors to identify genes that highly correlate with cell surface NIS protein levels. The heat maps are labeled with breast tumor IDs, molecular subtypes, and cell surface NIS protein levels corresponding to each breast tumor. Significance was assigned to genes with a correlation coefficient greater than 0.6 in conjunction with a p-value < 0.002. Sixty three genes were positively correlated (n = 44) or inversely correlated (n = 19) with NIS expression by Pearson correlation and 64 genes were positively (n = 42) or negatively (n = 22) correlated with cell surface NIS protein levels by Spearman rank correlation. High gene expression is shown in *red *and low gene expression is shown in *green*. (C) The box and whisker plots examine and compare normalized mRNA expression of two of the most highly correlated genes identified by both analyses, QRICH1 and ND6, among breast tumors scored as 0, 0/1+, 1+, 1+/2+, 2+ or 3+ for cell surface NIS protein. The length of the box represents the interquartile range (i.e., the middle 50% of the data). The median (*line through the middle of each box*), the lower quartile (*bottom line of each box*), and the upper quartile (*top line of each box*) are also specified on the plot for each level of cell surface NIS protein. The sample minimum and maximum values are represented as T-shaped lines extending from the ends of the box. Maximum outliers (*gray squares*) and minimum outliers (*black diamonds*) are also plotted. Click here for file
