# Supplementary figures and images for: Microarray analysis of genes associated with cell surface NIS protein levels in breast cancer
Source: BMC Res Notes. 2011 Oct 11;4:397. doi: 10.1186/1756-0500-4-397 (PMC3205061; doi:10.1186/1756-0500-4-397)

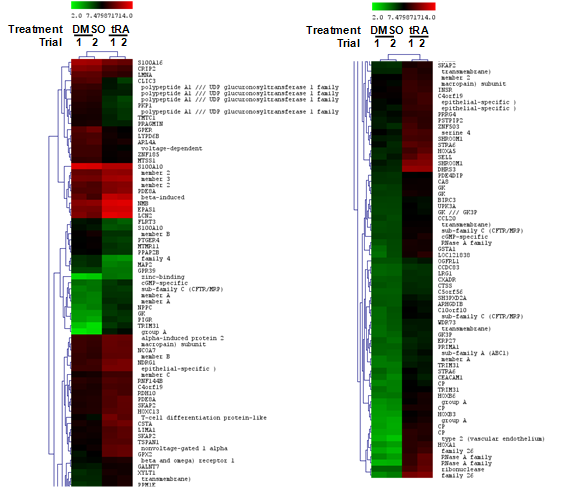

Supplement: Additional file 1 — Cluster analysis of 138 genes identified to be significantly up- or down-regulated with tRA treatment compared DMSO vehicle in MCF-7 cells, as determined by Linear Models analysis. MCF-7 human breast cancer cells were treated with DMSO vehicle or tRA (1 μM) for 12 hours, total RNA was harvested, genome-wide expression was detected by oligonucleotide microarray (Affymetrix HG U133 Plus 2.0) and genome-wide expression was compared between treatments by Linear Model analysis. Significance was assigned to 138 genes with a false discovery rate ≤0.045% and a p-value less than 0.00045. The heat map shows expression of these 138 genes in tRA- and DMSO vehicle-treated MCF-7 cells from two independent trials. Twenty eight genes were significantly up-regulated and 110 genes were significantly down-regulated in tRA-treated MCF-7 cells compared to DMSO vehicle control, although the NIS gene was not identified among them. Genes with high expression are denoted in red and genes with low expression are denoted in green. No genes were commonly identified by both the MCF-7 cell model and breast tumor analyses. [file 1756-0500-4-397-S1.TIFF]

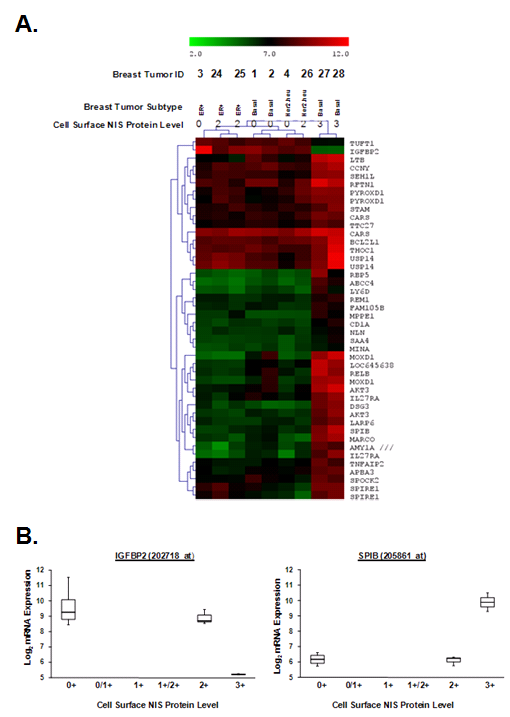

Supplement: Additional file 2 — Cluster analysis of 44 genes identified to be significantly up- or down-regulated in cell surface NIS-positive breast tumors compared to cell surface NIS-negative breast tumors by Linear Models analysis. The Linear Models analysis compared gene expression of 4 breast tumors considered to be negative for cell surface NIS protein (0+) to 5 tumors considered to be strongly positive for cell surface NIS protein (2+/3+). (A) Breast tumor IDs, molecular subtypes and cell surface NIS protein levels of breast tumors are indicated on the heat map. Significance was assigned to genes with a False Discovery Rate threshold of ≤0.038% and a p-value < 0.0004. The Linear Models analysis identified 42 genes to be significantly up-regulated and 2 genes to be significantly down-regulated in cell surface NIS-positive tumors compared to cell surface NIS-negative tumors. The cluster analysis shown in the heat map above appeared to cluster ER+ and HER-2/neu breast tumors according to molecular subtype and, within the ER+ molecular subtype, breast tumors appeared to group according to the level of cell surface NIS protein. In general, gene clusters capable of distinguishing between NIS-positive and NIS-negative ER+ and Her-2/neu breast tumors could not be identified. In contrast, cluster analysis distinguished between cell surface NIS-positive and cell surface NIS-negative basal breast tumors. High gene expression is denoted in red and low gene expression is denoted in green on the heat map. (B) The box and whisker plot examines and compares normalized mRNA expression of IGFBP2 and SPIB among breast tumors scored as 0, 0/1+, 1+, 1+/2+, 2+ or 3+ for cell surface NIS protein. The length of the box represents the interquartile range (i.e., the middle 50% of the data). The median (line through the middle of each box), the lower quartile (bottom line of each box), and the upper quartile (top line of each box) are also specified on the plot for each level of cell surface NIS protein. The sample [file 1756-0500-4-397-S2.TIFF]

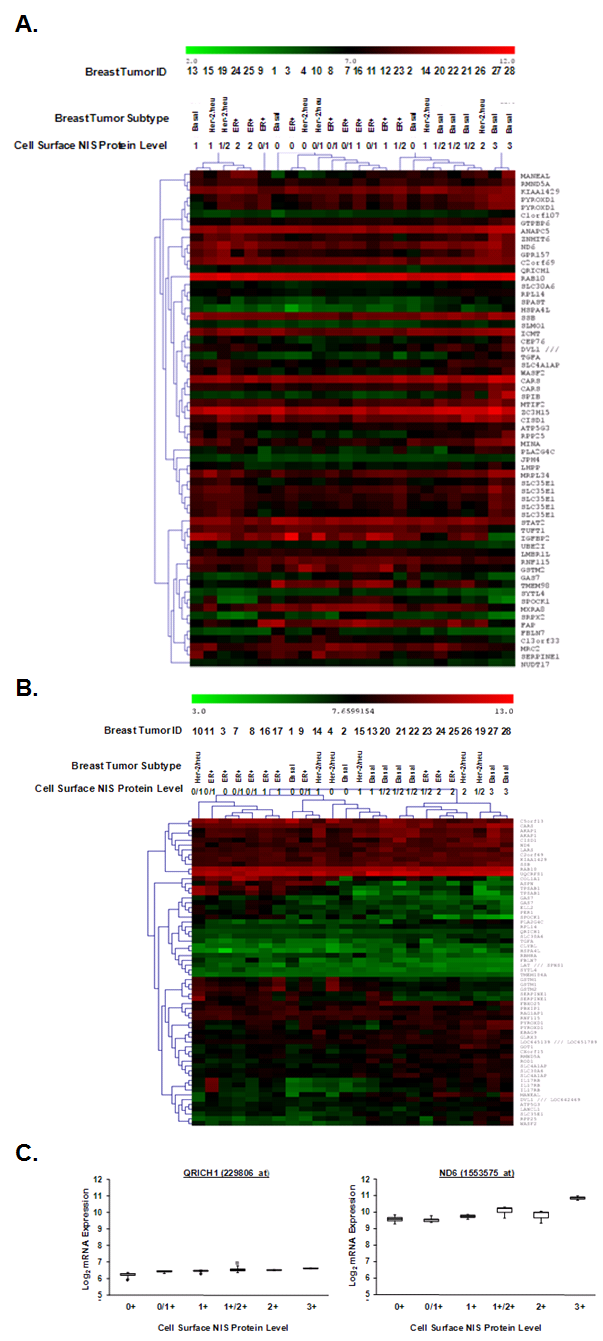

Supplement: Additional file 3 — Genes identified to be positively- or negatively-correlated with cell surface NIS protein levels in breast cancer. The (A) Pearson and (B) Spearman rank correlation analyses compared gene expression of 24 breast tumors to identify genes that highly correlate with cell surface NIS protein levels. The heat maps are labeled with breast tumor IDs, molecular subtypes, and cell surface NIS protein levels corresponding to each breast tumor. Significance was assigned to genes with a correlation coefficient greater than 0.6 in conjunction with a p-value < 0.002. Sixty three genes were positively correlated (n = 44) or inversely correlated (n = 19) with NIS expression by Pearson correlation and 64 genes were positively (n = 42) or negatively (n = 22) correlated with cell surface NIS protein levels by Spearman rank correlation. High gene expression is shown in red and low gene expression is shown in green. (C) The box and whisker plots examine and compare normalized mRNA expression of two of the most highly correlated genes identified by both analyses, QRICH1 and ND6, among breast tumors scored as 0, 0/1+, 1+, 1+/2+, 2+ or 3+ for cell surface NIS protein. The length of the box represents the interquartile range (i.e., the middle 50% of the data). The median (line through the middle of each box), the lower quartile (bottom line of each box), and the upper quartile (top line of each box) are also specified on the plot for each level of cell surface NIS protein. The sample minimum and maximum values are represented as T-shaped lines extending from the ends of the box. Maximum outliers (gray squares) and minimum outliers (black diamonds) are also plotted. [file 1756-0500-4-397-S3.TIFF]
